# miR-218-5p restores sensitivity to gemcitabine through PRKCE/MDR1 axis in gallbladder cancer

**DOI:** 10.1038/cddis.2017.178

**Published:** 2017-05-11

**Authors:** Hui Wang, Ming Zhan, Sun-Wang Xu, Wei Chen, Man-Mei Long, Yong-Heng Shi, Qiang Liu, Man Mohan, Jian Wang

**Affiliations:** 1 Department of Biliary-Pancreatic Surgery, Renji Hospital, School of Medicine, Shanghai Jiao Tong University, Shanghai, China; 2 Department of Pathology, Renji Hospital, School of Medicine, Shanghai Jiao Tong University, Shanghai, China; 3 Department of Biochemistry and Molecular Cell Biology, Shanghai Key Laboratory of Tumor Microenvironment and Inflammation, Institutes of Medical Sciences, Shanghai Jiao Tong University, School of Medicine, Shanghai 200025, China

## Abstract

Gallbladder cancer (GBC) is one of the most common malignancy of the biliary tract characterized by its high chemoresistant tendency. Although great progresses have been made in recent decades for treating many cancers with anticancer drugs, effective therapeutics methods for anti-GBC are still lacking. Therefore, investigations into identifying the mechanisms underlying the drug resistance of GBC are greatly needed. In this study, we show that miR-218-5p plays a critical role in gemcitabine resistance of GBC. miR-218-5p levels were significantly lower in GBC than adjacent non-cancer tissues, and which were also associated with patient prognosis. While miR-218-5p overexpression abrogated gemcitabine resistance of GBC cells, silencing of which exhibited the opposite effects. Via six microRNA targets prediction algorithms, we found that *PRKCE* is a potential target of miR-218-5p. Moreover, miR-218-5p overexpression repressed the luciferase activity of reporter constructs containing 3′-UTR of *PRKCE* and also reduced *PRKCE* expression. Further studies revealed that miR-218-5p promotes sensitivity of gemcitabine by abolishing PRKCE-induced upregulation of *MDR1*/P-gp. Taken together, our results imply that an intimate correlation between miR-218-5p and *PRKCE*/*MDR1* axis abnormal expression is a key determinant of gemcitabine tolerance, and suggest a novel miR-218-5p-based clinical intervention target for GBC patients.

Gallbladder cancer (GBC) accounts for about 40% of all biliary tract carcinomas, and is one of the most common malignancy of the biliary tract.^1, 2^ Although associated with poor prognosis, the mechanism underlying the aggressive malignancy of GBC remains largely unknown. Moreover, unlike several other tumors, it is highly resistant to the currently available standard adjuvant therapy, which makes its treatment even more challenging.^3^ Although several potential targets and signaling pathways underlying GBC chemoresistance have been revealed, the precise mechanisms are still elusive.^4, 5, 6^ Thus deeper unraveling the molecular mechanisms of drug resistance is of vital importance and urgently needed.

MicroRNAs (miRNAs), 22 nucleotides on average, are a group of evolutionarily conserved, small, endogenous, single-stranded non-coding RNAs.^7^ Through binding the 3′-untranslated regions (3′-UTRs) of their target genes, they could regulate specific genes expression at the posttranscriptional level. Multiple miRNAs have been found implicated in various kinds of cancers. miRNAs are capable of modulating cell proliferation, differentiation, metabolism, apoptosis, and thus actively participate in regulating tumorigenesis and tumor progression.^8, 9^ Evidence suggests that miRNA can modulate the therapeutic efficacy in several cancer types,^10^ but it is still unclear whether and how deregulated miRNAs are involved in the chemoresistance of GBC. miR-218-5p, a tumor suppressor miRNA, has been found to be downregulated in several cancer types such as GBC, cervical cancer, colon cancer, and prostate cancer.^11, 12, 13, 14^ In this study, we assessed the involvement of miR-218-5p in GBC chemosensitivity.

Permeability glycoprotein (P-gp), also known as multidrug resistance 1 (MDR1), is a well-known membrane transporter, which is able to transport various kinds of toxic substances and exerts a protective effect under physiological conditions. However, this function when utilized by cancer cells, may lead to the effluxing of many drugs and enhanced chemoresistance.^15^ PRKCE, a member of protein kinase C (PKC) family, is a serine- and threonine-specific protein kinase that actively participate in promoting drug resistance through phosphorylating a variety of protein targets, such as P-gp, ATF2, PI3K, Stat3, and Erk.^16, 17^

Here, using a genome-wide miRNA expression profiling in six pairs of GBC and the corresponding non-cancerous gallbladder (CNG) tissues, we found that miR-218-5p is significantly downregulated in GBC. Further, we show that miR-218-5p regulates gemcitabine sensitivity in GBC cells by simultaneously repressing *PRKCE* expression. Due to the ability of *PRKCE* to activate *MDR1*/P-gp, repression of miR-218-5p causes abolishment of *PRKCE* inhibition and leads to increased *MDR1*/P-gp levels, and then enhances gemcitabine resistance of GBC cells. Moreover, we show that miR-218-5p and *PRKCE* are prognostic markers in GBC patients receiving chemotherapy. This adds to the possibility that strengthening of GBC chemoresistance might be countered by restoring expression of miR-218-5p, a notion that can be tested in the clinic.

## Results

### Reduced miR-218-5p expression in GBC well correlated with tumor prognosis

**Figure 1:**
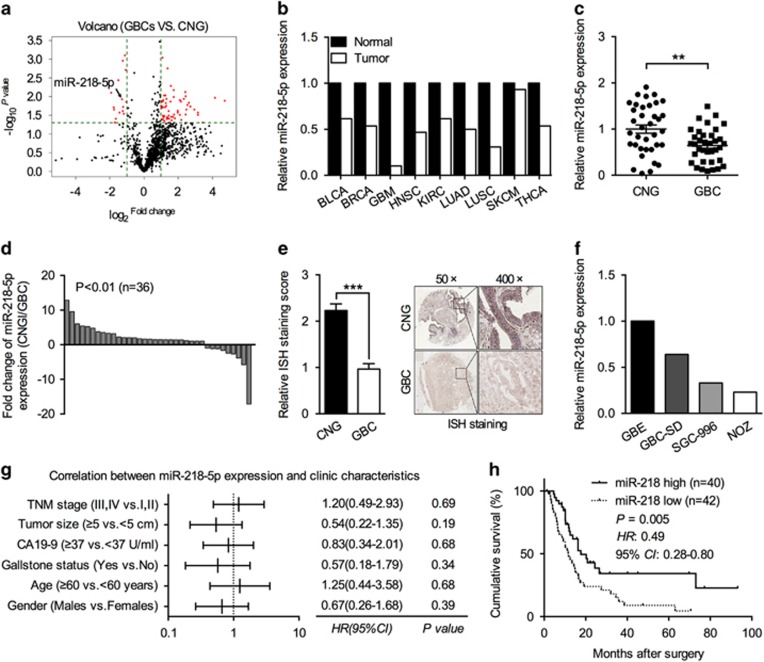
miR-218-5p is downregulated in GBC tissues and cell lines, and is associated with prognosis of GBC patients. (**a**) Volcano plot of microRNAs expression array from six pairs of GBC and CNG tissues. More detailed information can be found in Supplementary Materials. (**b**) miR-218-5p expression in other nine types of cancer from starBase v2.0 Pan-cancer Project database. (**c**) Validation of miR-218-5p differential expression in an independent cohort contained 36 pairs of GBC and CNG tissues. (**d**) Fold change of miR-218-5p expression in new 36 pairs of GBC and CNG tissues. (**e**) Comparison of miR-218-5p expression in 82 pairs of GBC and CNG FFPE tissues (left) and their representative ISH image stained by anti-miR-218-5p probe (right). (**f**) Relative expression of miR-218-5p in GBC cells and GBE cells. (**g**) Comparing different TNM stages, tumor size, CA19-9 level, gallstone status, age and gender ratio between miR-218-5p high and miR-218-5p low expression from 82 pairs of GBC FFPE tissues. Statistical significance was performed by the *χ*^2^-test. (**h**) Kaplan–Meier analysis of the correlation between miR-218-5p expression and POS in 82 GBC patients. Small nuclear RNA *U6* was used to normalize the Q-PCR results. bar, S.E.M., **P<0.01; ***P<0.001; Student’s *t*-test. BLCA, urothelial bladder cancer; BRCA, breast cancer; GBM, glioblastoma multiforme; HNSC, head and neck squamous cell carcinoma; KIRC, clear cell kidney carcinoma; LUAD, lung adenocarcinoma; LUSC, lung squamous cell carcinoma; SKCM, cutaneous melanoma; THCA, papillary thyroid carcinoma

To identify transcripts that potentially drive malignance of GBC, a miRNA expression profile was determined by microarray analysis. The volcano plot and heat map showed systematic variations in transcript expression levels of miRNAs between GBC tissues and CNG tissues from six GBC patients (Figure 1a, Supplementary Figure 1, and Supplementary Table 1). Further analyzing via our miRNA expression profile data and other microarray databases (starBase v2.0; http://starbase.sysu.edu.cn/) of different tumors,^18^ we found that miR-218-5p is one of the common target reduced in many kinds of tumors (Figure 1b and Supplementary Figure 2). For validating the possible involvement in GBC, we further assessed its expression in 36 pairs of fresh GBC tissues and CNG tissues. Indeed, reduced miR-218-5p expression was detected in GBC tumors samples (Figures 1c and d). *In situ* hybridization (ISH) staining also confirmed a remarkably lower miR-218-5p expression in GBC paraffin sections (Figure 1e). Moreover, we also noticed a reduced miR-218-5p expression in different GBC cell lines compared with normal gallbladder epithelial cell (GBE) cells (Figure 1f). Intriguingly, further correlation analysis manifested no obvious relationship between miR-218-5p expression and patients’ clinic characteristics such as TNM stage, tumor size, CA 19-9 level, gallstone status, age, and gender (Figure 1g; Supplementary Table 2). However, an inverse relationship between miR-218-5p and cumulative survival rate was observed (Figure 1h).

### Enforcing miR-218-5p expression accelerated tumor cell death and improved chemotherapeutic efficacy

**Figure 2:**
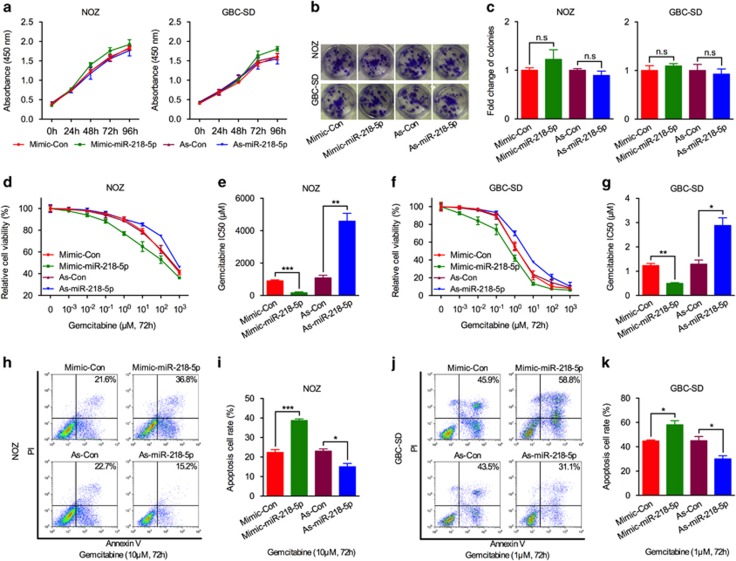
miR-218-5p promotes sensitivity of gemcitabine in NOZ and GBC-SD cell lines. (**a**) MTS analysis of GBC cells (NOZ and GBC-SD) transiently transfected with miR-218-5p mimic (Mimic-miR-218-5p), negative control mimic (Mimic-Con), miR-218-5p antisense (As-miR-218-5p), or negative control antisense (As-Con). (**b**–**c**) Representative images of colony formation and the mean fold change of colonies induced by miR-218-5p mimic or antagomir in GBC cells. (**d**–**g**) MTS assay and calculating the corresponding IC50 of gemcitabine in GBC cells transfected with miR-218-5p mimic or antagomir. (**h**–**k**) Flow cytometric analysis of Annexin V/PI staining used to quantify apoptosis rate of GBC cells transfected with miR-218-5p mimic or antagomir, followed by exposure to gemcitabine (NOZ, 10 *μ*M; GBC-SD, 1 *μ*M) for 72 h. All *n*=3; bar, S.E.M., NS, not significant, *P<0.05; **P<0.01; ***P<0.001; Student’s *t*-test

To further explore the biological effect of miR-218-5p, we overexpressed and reduced miR-218-5p in GBC cell lines (NOZ and GBC-SD) by transfecting miR-218-5p mimics and antisense, respectively. However, no obvious difference in proliferation and colony formation ability was found (Figures 2a–c). GBC is well known for its higher chemoresistance than other kinds of tumors. Therefore, we wondered if miR-218-5p expression influences GBC patients’ prognosis through influencing the chemotherapeutic efficacy. To verify this hypothesis, we treated miR-218-5p overexpressing or downregulated NOZ and GBC-SD cell lines with various concentrations of gemcitabine and analyzed the viability at 72 h post treatment. Surprisingly, enforced miR-218-5p expression improved the antitumor effect of gemcitabine in NOZ and GBC-SD cells, with a significant reduction in IC50. In contrast, reduced miR-218-5p expression enhanced gemcitabine resistance ability, with a remarkable increase in IC50 (Figures 2d–g). To validate these results, the effect of miR-218-5p was examined by flow cytometry analysis of Annexin V positive cells after gemcitabine addition. Consistently, similar results were obtained showing that miR-218-5p overexpression greatly accelerated cell death, and downregulation inhibited cell apoptosis after gemcitabine treatment (Figures 2h–k). However, miR-218-5p itself has no effect on the apoptosis of GBC cells (Supplementary Figure 3). In conclusion, our results demonstrate that miR-218-5p can sensitize GBC cells to chemotherapeutic treatment.

### miR-218-5p targets 3′-UTR of PRKCE and suppresses its expression

**Figure 3:**
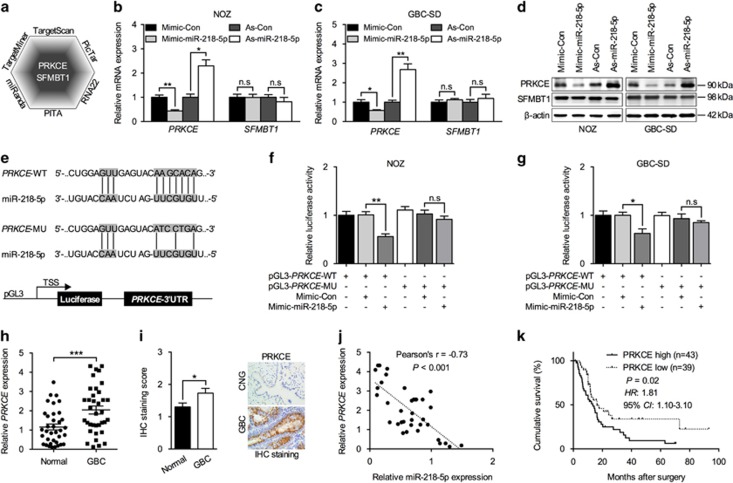
miR-218-5p directly targets the 3′-UTR of *PRKCE* and downregulated its expression. (**a**) *PRKCE* and *SFMBT1* were potential targets of miR-218-5p in all six miRNA target prediction algorithms. (**b**, **c**) Q-PCR to detect *PRKCE* and *SFMBT1* mRNA level in GBC cells when transfected with miR-218-5p mimic or antagomir. *n*=3; bar, S.E.M. (**d**) Western blot to analyze *PRKCE* and *SFMBT1* protein levels in GBC cell lines when transfected with miR-218-5p mimic or antagomir. (**e**) A schematic diagram showing the predicted miR-218-5p binding sites and the designed mutant sequence in the 3′-UTR of *PRKCE* (up), and the luciferase reporter constructs (down). (**f**, **g**) Firefly luciferase activity analysis of *PRKCE* 3′-UTR performed after co-transfection with *PRKCE*-wild type or *PRKCE*-mutant pGL3 constructs and miR-218-5p mimic GBC cell lines. *n*=3; bar, S.E.M. (**h**) Q-PCR analysis of *PRKCE* mRNA levels in 36 pairs of GBC and CNG tissues. (**i**) Semi-quantitative analysis and the representative images (× 400) of IHC staining for *PRKCE* protein in 82 paired GBC and CNG FFPE tissues. (**j**) The correlation between miR-218-5p and *PRKCE* expression in 36 GBC tissues measured by Q-PCR. (**k**) POS analysis based on *PRKCE* protein expression levels in 82 GBC patients. *GAPDH* was used to normalize the Q-PCR results, and *β*-actin was the loading control in western blot assay. NS, not significant, *P<0.05; **P<0.01; ***P<0.001; Student’s *t*-test

miRNA usually regulate specific gene expression by binding the 3′-UTR of their target genes. Through prediction analysis using six different databases, two common targets of miR-218-5p were found (*PRKCE* and *SFMBT1*) (Figure 3a). We then analyzed the mRNA expression of *PRKCE* and *SFMBT1* in miR-218-5p mimics or antisense-transfecting NOZ and GBC-SD cells. Surprisingly, only *PRKCE* showed reduced expression in miR-218-5p overexpressing cells, and increased expression upon miR-218-5p downregulation (Figures 3b and c). No obvious alterations in *SFMBT1* mRNA expression were observed (Figures 3b and c). Further, we analyzed *PRKCE* and *SFMBT1* protein expression by immunoblotting analysis, and found that miR-218-5p reduced *PRKCE* protein expression in both NOZ and GBC-SD cells. No obvious alterations in the *SFMBT1* protein amount were detected (Figure 3d). We then mutated the predicted complementary paring region of the 3′-UTR of *PRKCE*-WT (5′-AAGCACA-3′) to *PRKCE*-MU (5′-ATCCTGA-3′) (Figure 3e), and cloned into luciferase reporter vector. miR-218-5p addition reduced the luciferase activity carrying *PRKCE*-WT reporter but failed to do so with *PRKCE*-MU transfection in NOZ cells (Figure 3f). Similar results were obtained in GBC-SD cells (Figure 3g). *PRKCE* has been previously suggested to be involved in multiple kinds of tumors, but its involvement in GBC has never been investigated. We analyzed *PRKCE* expression in our 36 pairs of CNG and GBC tumor samples, and found an increased expression in GBC (Figure 3h). Immunohistochemistry (IHC) staining further corroborated our results (Figure 3i). Interestingly, an inverse correlation between miR-218-5p and *PRKCE* mRNA expression was detected in GBC samples (Figure 3j). Moreover, higher PRKCE expression correlated with reduced cumulative survival rate, implying a poorer prognosis with PRKCE overexpression (Figure 3k).

### The chemotherapeutic sensitizer function of miR-218-5p is PRKCE dependent

**Figure 4:**
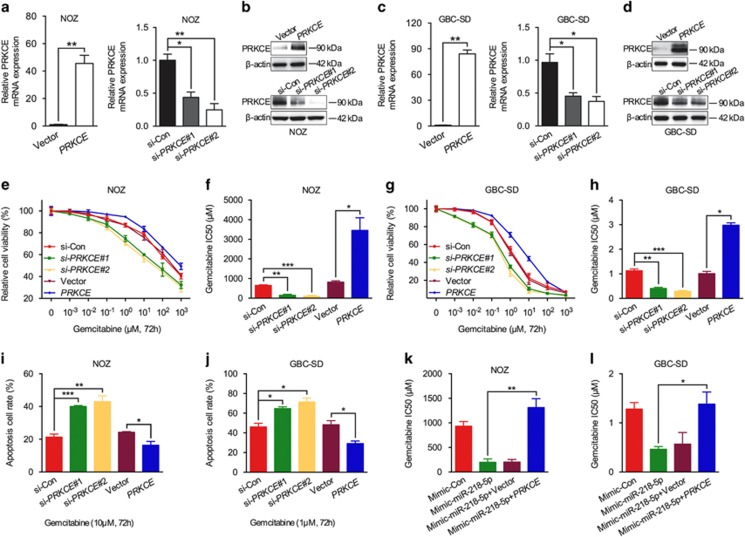
miR-218-5p-sensitized GBC cells to gemcitabine was mediated by PRKCE. (**a–d**) Efficiency testing of overexpression or interference of *PRKCE* in GBC cells by using Q-PCR and western blot. (**e-h**) MTS assay and calculating the corresponding IC50 of gemcitabine in GBC cells transfected with *PRKCE* construct or *PRKCE* siRNAs. (**i**, **j**) Flow cytometric analysis of Annexin V/PI staining used to quantify apoptosis rate of GBC cells transfected with *PRKCE* construct or *PRKCE* siRNAs, followed by exposure to gemcitabine (NOZ, 10 *μ*M; GBC-SD, 1 *μ*M) for 72 h. (**k**, **l**) IC50 of gemcitabine in GBC cells co-transfected with miR-218-5p mimic and *PRKCE* construct. *GAPDH* was used to normalize the Q-PCR results, and *β*-actin was the loading control in western blot assay. All *n*=3; bar, S.E.M. *P<0.05; **P<0.01; ***P<0.001; Student’s *t*-test

To find out whether *PRKCE* is involved in regulating chemosensitivity of GBC, we overexpressed and knocked down *PRKCE* in GBC cell lines using overexpression vector and siRNA, respectively. Quantitative-PCR (Q-PCR) and immunoblotting analysis confirmed the overexpression and knock down efficiency of *PRKCE* (Figures 4a–d). Indeed, downregulation of *PRKCE* expression reduced IC50 of gemcitabine and increased tumor-killing effect of gemcitabine in NOZ and GBC-SD cells. On the other hand, *PRKCE* overexpression had the opposite effect and remarkably increased IC50 of gemcitabine, and enhanced chemoresistance (Figures 4e–j). Next for confirming *PRKCE* the potential downstream effector executing the anti-therapy effect with miR-218-5p deficiency, *PRKCE* were overexpressed in concomitant with miR-218-5p mimic transfection in NOZ and GBC-SD cell lines in parallel. As expected, *PRKCE* overexpression abolished the increased efficacy of gemcitabine with the addition of miR-218-5p mimic (Figures 4k–l). Our results demonstrate that miR-218-5p directly targeted *PRKCE* and involved in the chemotherapeutic efficacy.

### miR-218-5p/PRKCE targeted MDR1 involved in the chemoresistance of GBC

**Figure 5:**
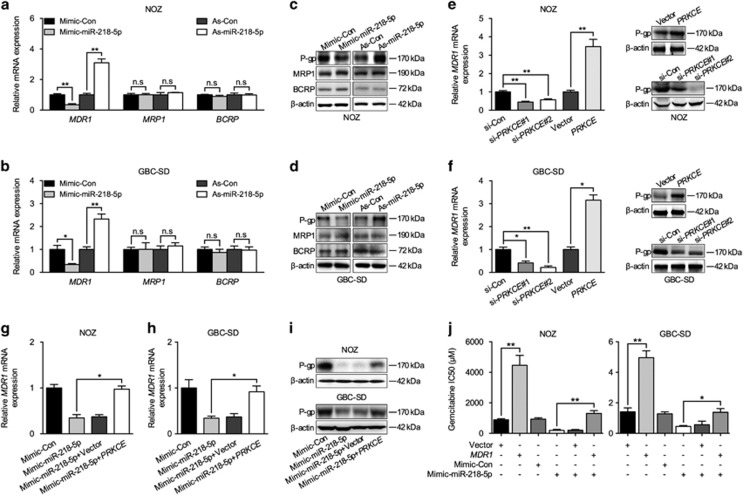
miR-218-5p negatively regulates resistance to gemcitabine through PRKCE/MDR1 axis in GBC cells. (**a–d**) Q-PCR and western blot to analyze *MDR1*, *MRP1*, and *BCRP* mRNA and protein expression, respectively, in GBC cell lines when transfected with miR-218-5p mimic or antagomir. (**e**, **f**) Q-PCR and western blot to analyse *MDR1* mRNA and P-gp expression, respectively, in GBC cell lines when transfected with *PRKCE* construct or *PRKCE* siRNAs. (**g–i**) Q-PCR and western blot to detect *MDR1* mRNA and P-gp expression, respectively, in GBC cell lines when co-transfected with miR-218-5p mimic and *PRKCE* construct. (**j**) IC50 of gemcitabine in GBC cells when co-transfected with miR-218-5p mimic and *MDR1* construct. *GAPDH* was used to normalize the Q-PCR results, and *β*-actin was the loading control in western blot assay. All *n*=3; bar, S.E.M. *P<0.05; **P<0.01; Student’s *t*-test

Our previously study showed that *MDR1*, *MRP1*, *BCRP* are involved in the chemoresistance mechanisms of GBC.^4, 5, 6^ Several studies have implicated PKC family in regulating several chemotherapeutic-resistant protein such as P-gp and BCRP. In order to find out whether miR-218-5p is also capable of modulating these proteins, *MDR1*, *MRP1*, *BCRP* mRNA, and protein expression were analyzed in NOZ and GBC-SD cells transfected with miR-218-5p mimic or antisense by Q-PCR and western blot. Among these three genes, only *MDR1*/P-gp exhibited an altered expression. miR-218-5p overexpression inhibited *MDR1*/P-gp expression, while reduced miR-218-5p increased *MDR1*/P-gp expression (Figures 5a–d). On the other hand, our results also showed that *MDR1*/P-gp expression was regulated by *PRKCE* in NOZ and GBC-SD cells. Knockdown of *PRKCE* decreased *MDR1*/P-gp levels, whereas overexpression of *PRKCE* increased *MDR1*/P-gp expression (Figures 5e–f). Interestingly, when *PRKCE* is overexpressed simultaneously with miR-218-5p mimic, *MDR1*/P-gp expression was restored (Figures 5g–i). In addition, *MDR1* overexpression not only strengthened the chemoresistance of GBC cells to gemcitabine, but also abrogated the effect of miR-218-5p overexpression on promoting gemcitabine sensitivity as shown by restored IC50 (Figure 5j). All the above results demonstrated that miR-218-5p-*PRKCE*-*MDR1* axis is a potential candidate target in GBC chemotherapeutic treatment.

### The improved chemotherapeutic efficacy of miR-218-5p addition *in vivo*

**Figure 6:**
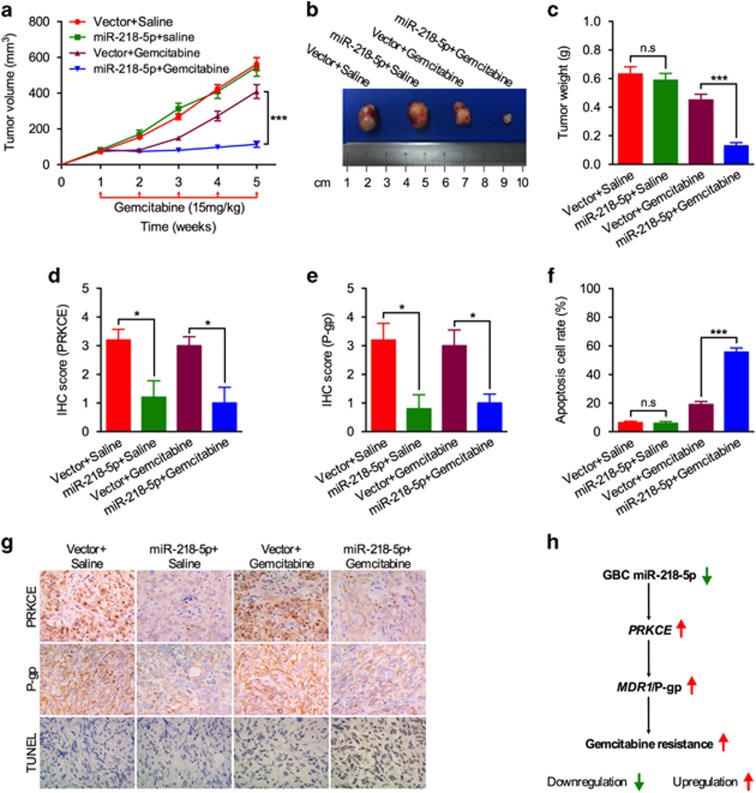
Effect of miR-218-5p overexpression on the gemcitabine sensitivity of GBC cells *in vivo*. (**a**) Tumor growth curves of NOZ cells transfected with miR-218-5p construct or empty vector treated with gemcitabine or saline *in vivo*. (**b**, **c**) Representative images and the mean tumor weight of the four paired groups formed at the 5th week after subcutaneous transplantation. (**d–f**) Semi-quantitative analyses of IHC results for PRKCE and P-gp expression, and percentage of apoptotic tumor cells from the four paired NOZ tumor xenografts. (**g**) Representative images of PRKCE and P-gp IHC staining, and TUNEL staining in paraffin sections from the four paired NOZ tumor xenografts (× 400). (**h**) Schematic representation of pathway modulated by miR-218-5p in GBC cells. Downregulation of miR-218-5p in GBC cells can elevate PRKCE expression. This increases the expression of *MDR1*/P-gp, resulting in gemcitabine resistance of GBC. All *n*=5; bar, S.E.M. NS, not significant, *P<0.05; ***P<0.001; Student’s *t*-test

In order to confirm the relationship of miR-218-5p with chemotherapeutic effect *in vivo*, we utilized a tumor xenograft mouse model. NOZ cells were stably transfected with a miR-218-5p plasmid construct or control plasmid and subcutaneously injected into male nude mice. A week later, one group was treated with gemcitabine (15 mg/kg per week) administered by intraperitoneal administration, and the other with the carrier (Saline). Consistently, miR-218-5p addition had no effect on tumor size in mouse group with no drug treatment (Figures 6a–c). However, tumor sizes and weight were greatly reduced in miR-218-5p overexpression mouse treated with gemcitabine (Figures 6a–c). IHC analysis of PRKCE and P-gp both exhibited reduced expression with miR-218-5p overexpression (Figures 6d–e, g). Transferase dUTP nick end-labeling (TUNEL) analysis of tumor specimens showed that the antitumor effect of gemcitabine was greatly enhanced in miR-218-5p overexpression mouse due to increased apoptosis (Figures 6f–g). These results from tumor xenograft mouse model provided further evidence that miR-218-5p enhances gemcitabine-induced apoptosis by targeting *PRKCE* and *MDR1*.

## Discussion

The high chemoresistance of GBC made its treatment even more difficult and prognosis more pessimistic. Our study identified a novel miR-218-5p-*PRKCE*-*MDR1* pathway implicated in modulating GBC chemosensitivity (Figure 6h). Reduced miR-218-5p expression in GBC, disabled its ability in degrading PRKCE, which eventually led to increased *MDR1*/P-gp expression. P-gp, one of the MDR-related proteins, played important role in orchestrating chemoresistant effect of gemcitabine.^19, 20^ Thus, future treatment targeting miR-218-5p-*PRKCE*-*MDR1* pathway would provide promising prospect for GBC patients.

Increasing evidence suggests that miR-218-5p exhibits antitumor effect in the development and progression of various human cancers through influencing tumor proliferation, invasion, and metastasis progression.^11, 12, 13, 21^ Moreover, recent studies suggest that miR-218-5p also exert critical role on regulating chemosensitivity.^22, 23, 24^ For example, miR-218-5p improved 5-fluorouracil therapeutic efficacy through inhibiting *BIRC5* and *TS* expression, both of which are important contributors to chemoresistance.^22^ Another investigation in glioblastoma multiforme cells found that elevating miR-218-5p increased sensitivity to cisplatin via inactivating RTK-HIF2*α* signaling axis.^23^ More remarkably, altered miR-218-5p expression in breast cancer cells modulated the sensitivity of cisplatin through regulating BRCA1 expression.^24^ However, whether and how miR-218-5p might be involved in GBC has not been studied. Consistent with previous studies, we also found a significantly reduced miR-218-5p expression of GBC tissues compared with CNG tissues and correlated with a poor prognosis. Simultaneously, an increased sensitivity to gemcitabine was observed *in vitro* miR-218-5p overexpression. But no obvious effects on GBC cell growth and apoptosis were detected. This implied that miR-218-5p specifically influenced GBC chemosensitivity and thus changed GBC patients’ prognosis.

So far, miR-218-5p has been reported to bind to various targets such as *ARAF*, *PIK3C2A*, *LASP1*, *BRCA1*, *SFMBT1*, *DCUN1D1*, *and PXN* to repress corresponding gene translation.^12, 13, 23, 24, 25, 26^ However, the function of miR-218-5p and the association of its target gene with gemcitabine resistance in GBC remains unclear. Further elucidating the precise mechanism inside, *in silico* analysis of putative miRNA-binding sites in 3′-UTR of the target genes by using different algorithms (TargetScan, PicTar, RNA22, PITA, miRanda, and TargetMiner) was performed. *PRKCE* and *SFMBT*1 were finally identified, both harboring evolutionarily conserved targeting sequence of miR-218-5p. Facilitated by further *in vitro* overexpression and site mutation-related luciferase assay, we then confirmed the regulatory role of *PRKCE* by miR-218-5p in GBC tumors. Emerging evidences suggested that *PRKCE* plays oncogenic role through increasing survival protein levels, which sustained cancer stem cell development.^17^ Our study reinforced the oncogenic role of *PRKCE* in increasing gemcitabine resistance and its correlation with a poor prognosis in GBC tumors. The inverse relationship between miR-218-5p and *PRKCE* was then confirmed in GBC tissues. Therefore, early examination of miR-218-5p and *PRKCE* expression in the tumors of GBC patients may aid in the prediction of chemotherapeutic effect and help in the development of new postoperative chemotherapy strategies. However, preclinical and clinical studies with large sample sizes and longer follow-up times are required to confirm the utility of these potential biomarkers.

Reducing intracellular drug accumulation is one of the most important mechanisms utilized by cancer cells in chemoresistance. Elevated drug efflux pump-related proteins such as P-gp, MRP1, MRP2, and BCRP expression significantly promotes cancer chemoresistance.^27^ Consistent with the previous report in renal carcinoma cells that reduce the expression of *PRKCE* lowered *MDR1*/P-gp, which affects the cancer stem cell potential of sorted side population cells and suppresses proliferation potential, resistance to chemotherapeutics and *in vivo* tumor formation ability.^17^ Our identification of the regulatory role of miR-218-5p-*PRKCE*-*MDR1* axis in GBC cells further confirmed its importance.

Taken together, our study demonstrated that miR-218-5p is a potent tumor chemoresistance suppressor in the GBC and that its chemosensitization effects are mediated, at least in part, via downregulation of the PRKCE/MDR1 pathway. Loss of miR-218-5p expression, leading to the induction of *PRKCE* and its downstream *MDR1*/P-gp expression, appears to be a critical event in the development of gemcitabine resistance.

## Materials and Methods

### Patients and tissue samples

This study was approved by the Ethical Committee of Renji hospital, Shanghai Jiao Tong University School of Medicine. A total of 82 pairs of formalin-fixed, paraffin-embedded (FFPE) GBC tissues and CNG tissues were retrieved from GBC patients who underwent surgical resection and postoperative adjuvant chemotherapy from the Department of Pathology of Renji Hospital. Among the 82 GBC patients, 36 pairs of fresh GBC tissues and CNG tissues were also collected after the surgical removal and snap-frozen in liquid nitrogen immediately, then stored at −80 °C until total RNA was extracted. All 82 GBC patients were retrospectively followed up until December 2014. Postoperative survival (POS) was defined as the interval between the dates of surgery and last follow-up or death. All the subjects provided written informed consent in this study.

### Cell culture

Three different kinds of human GBC cell lines were used in this study. GBC-SD was purchased from the Cell Bank of Type Culture Collection of Chinese Academy of Sciences (Shanghai, China). SGC-996 was provided by the academy of life sciences, Tong Ji university (Shanghai, China). NOZ were obtained from the Health Science Research Resources Bank (Osaka, Japan). GBC-SD, SGC-996, and NOZ were cultured in DMEM, RPMI-1640, and William’s E medium (Gibco, Grand Island, NY, USA), respectively, which supplemented with 10% fetal bovine serum (FBS) in a humidified atmosphere of 5% CO_2_ at 37 °C. All cell lines were ensured mycoplasma-negative cultures by monthly mycoplasma tests. For primary GBE culturing, cells from digested gallbladder epithelial tissue were cultured in KSFM medium without FBS. After 30 min, adherent cells were discarded, and non-adherent cells were continually cultured at 37 °C in 5% humidified CO_2_.

### Cell transfection

The miR-218-5p mimic and a non-specific mimic control, miR-218-5p antisense and a non-specific antisense control, *PRKCE* siRNA, and a negative control siRNA were all purchased from GenePharma (Shanghai, China), and were transfected into GBC cell lines by using Lipofectamine 2000 reagent (Invitrogen, Carlsbad, CA, USA) according to instructions of the manufacturer. Human miR-218-5p expression construct was generated by insertion of the coding sequence (CDS) of miRNA into pCDH-CMV-MCS-EF1-copGFP (System Biosciences, Palo Alto, CA, USA). Then recombinant lentiviruses produced in HEK293FT cells infect GBC cell lines in the presence of 4 *μ*g/ml polybrene (Sigma, St. Louis, MO, USA), followed by puromycin selection (2 *μ*g/ml) for stably overexpressing GBC cell lines. *PRKCE* and *MDR1* overexpression vector was generated by insertion of gene CDS into a pcDNA 3.1 vector (Invitrogen). GBC-SD and NOZ cells were transfected with the overexpression plasmid using Lipofectamine 2000 reagent. The pCDH-CMV-MCS-EF1-copGFP and pcDNA 3.1 empty vector were used as negative control (vector), respectively.

### Cloning efficiency assays

For colony formation, cells in single-cell suspension were plated and grown in 24-well plates at a density of 100 per well for 24 h. After transfecting, GBC cells were incubated in the plate for 14 days until colonies were visible. The colonies were fixed for 15 min with 4% paraformaldehyde and stained with 0.1% crystal violet. The numbers of colonies were counted to assess the viability.

### Cell viability and apoptosis assays

After transfecting with miRNA mimic or antisense and treating with concentration gradient of gemcitabine (Selleck, Houston, TX, USA), the viability of GBC cell was measured using 3-(4,5-dimethylthiazol-2-yl)-5-(3-carboxymethoxyphenyl)-2-(4-sulfophenyl)-2H-tetrazolium (Promega, Madison, WI, USA) assay (MTS), as described previously.^4^ After transfecting with gemcitabine for 48 h, cells were stained with FITC-conjugated Annexin V (BD Biosciences, Heidelberg, Germany) and propidium iodide (5 mg/ml) and analyzed by fluorescence-activated cell sorting analysis, as described previously.^4^

### Immunoblot analysis

Total proteins were extracted from GBC cells using cell lysates in RIPA buffer and protein expression was assessed by immunoblot analysis according to the procedure described previously. The primary mouse, rabbit, or goat antibodies used were as follows: PRKCE (1:500, sc-1681, Santa Cruz, Santa Cruz, CA, USA), SFMBT1 (1:500, ab77419, Abcam, Cambridge, UK), MDR1/P-gp (1:500, sc-55510, Santa Cruz), MRP1 (1:2000, sc-365635, Santa Cruz), BCRP (1:200, sc-25822, Santa Cruz), and *β*-actin (1:2000, A5316, Sigma).

### Quantitative real-time PCR analysis

TRI reagent (Sigma) was used to isolate total RNA for mRNA analysis, and miRNeasy kit (Qiagen, Valencia, CA, USA) for miRNA analysis from the snap-frozen tissues or cultured cells. After determining RNA concentration and purity by using NanoDrop ND-8000 (Thermo Fisher Scientific, Waltham, MA, USA), the cDNAs were synthesized by using Reverse Transcriptase M-MLV kit (Invitrogen). The expression levels of miRNA and mRNA were analyzed using SYBR Premix Ex Taq (Takara, Shiga, Japan) in Applied Biosystems ViiATM 7 Real-Time PCR System (Applied Biosystems, Foster City, CA, USA). Data were analyzed with 2^−ΔΔCT^ method^28^ for quantification of the relative mRNA expression levels. Expression values of genes and miRNA were first normalized against *GAPDH* and small nuclear *U6* RNA, and then compared to experimental controls. The primers were purchased from Sangon Biotech (Shanghai, China) and the sequences are listed in Supplementary Table 3.

### Immunohistochemistry and terminal deoxynucleotidyl transferase dUTP nick end-labeling assays

The IHC and TUNEL staining of FFPE tissue and its scoring system were performed, as described previously.^4^ The mouse primary antibodies used were as follows: PRKCE (1:200, sc-1681, Santa Cruz), MDR1/P-gp (1:200, sc-55510, Santa Cruz).

### Dual-luciferase reporter assay

The 3′-UTR of *PRKCE* containing the predicted miR-218-5p binding site was amplified by PCR using cDNA of GBC cell as a template and then subcloned into a pmirGLO Dual-Luciferase miRNA Target Expression Vector (Promega) to form the reporter vector MRP1-3′-UTR wild type. The mutant 3′-UTR of *PRKCE*, which contained point-mutated sequence in the binding region of miR-218-5p, was generated by using a site-directed mutagenesis kit from Fast Mutagenesis System (TransGen Biotech, Beijing, China). NOZ and GBC-SD cells were seeded into 96-well plates (1 × 10^4^ cells per well) and then co-transfected with miR-218-5p mimics and the luciferase reporter construct. After transfection for 48 h, the Renilla and firefly luciferase activities were analyzed using the Dual-Luciferase Assay System (Promega). The results of firefly luciferase activity were normalized to the Renilla luciferase activity.

### Bioinformatics

The target genes of miR-218-5p were predicted by six computer-aided algorithms,^29, 30, 31, 32^ namely TargetScan Release 7.0 (http://www.targetscan.org/vert_71/), PicTar (http://www.pictar.org/cgi-bin/new_PicTar_vertebrate.cgi), RNA22 (https://cm.jefferson.edu/rna22/), PITA (https://genie.weizmann.ac.il/pubs/mir07/mir07_prediction.html), miRanda (http://www.microrna.org/microrna/home.do), and Targetminer (http://www.isical.ac.in/~bioinfo_miu/targetminer20.htm). The target genes were accepted only when they were positive in all six analyses.

### *In situ* hybridization

ISH of GBC FFPE tissues were performed using a human miR-218-5p-specific digoxigenin-labeled locked nucleic acid probe. Briefly, following dewaxing and rehydration, tissue sections were treated with proteinase K at 37 °C for 15 min. We then washed specimens with PBS and dehydrated through a graded series of ethanol (70, 96 and 100%). After incubation under 50 °C overnight with miR-218-5p probe in hybridization buffer, the slides were washed in pre-warmed 5 ×, 1 × and 0.2 × SSC at 50 °C for 30 min. The slides were blocked with blocking reagent (Roche, Mannheim, Germany) and then incubated with antibody against digoxigenin (1:1000, Roche) at room temperature for 30 min, respectively. Finally, the substrate NBT/BCIP (Roche) was added on specimens and incubated for 15 min in dark until the specific blue signal was observed followed by stopping further reaction using KTBT buffer.

### Tumor formation assay in a nude mouse model

Twenty male BALB/c nude mice (4 weeks old; 15–25 g) were randomly divided into two groups. A total 2 × 10^6^ NOZ/pCDH empty vector and NOZ/pCDH miR-218-5p overexpression cells in 100 *μ*l medium were subcutaneously transplanted into the mouse left flank of two groups, respectively. One week later, each group was randomly divided into two subgroups (*n*=5) and subjected to intraperitoneal injection of gemcitabine (15 mg/kg) or saline (100 *μ*l; negative control) weekly. Tumor growth was measured using external caliper once every week and were calculated based on the equation: *V*=(length × width^2^)/2.^33^ All mice were killed at the 5th week, and the tumors were dissected out for IHC and TUNEL staining. The study was strictly performed accordance with the recommendations in the Guide for the Care and Use of Laboratory Animals of Shanghai Jiao Tong University.

### Statistics

Data were expressed as mean±S.E.M. Group comparisons of normally distributed data were performed with unpaired Student’s *t*-test (two-tailed) or one-way ANOVA. For multiple comparisons, the Tukey–Kramer honestly significant difference was applied following ANOVA. Kaplan–Meier analyses log-rank test were used to determined POS. The Pearson *χ*^2^-test was used to analyze the association of miR-218-5p expression with *PRKCE* expression. Dichotomous variables were compared using *χ*^2^-test. SPSS17.0 software (IBM, Chicago, IL, USA) was used for all statistical analysis. *P*<0.05 was considered statistically significant.

## References

[bib1] Castro FA, Koshiol J, Hsing AW, Devesa SS. Biliary tract cancer incidence in the United States-demographic and temporal variations by anatomic site. Int J Cancer 2013; 133: 1664–1671. 23504585 10.1002/ijc.28161 PMC3713194

[bib2] de Groen PC, Gores GJ, LaRusso NF, Gunderson LL, Nagorney DM. Biliary tract cancers. N Engl J Med 1999; 341: 1368–1378. 10536130 10.1056/NEJM199910283411807

[bib3] Lazcano-Ponce EC, Miquel JF, Munoz N, Herrero R, Ferrecio C, Wistuba II et al. Epidemiology and molecular pathology of gallbladder cancer. CA Cancer J Clin 2001; 51: 349–364. 11760569 10.3322/canjclin.51.6.349

[bib4] Zhan M, Wang H, Chen T, Chen W, Yang L, He M et al. NOX1 mediates chemoresistance via HIF1alpha/MDR1 pathway in gallbladder cancer. Biochem Biophys Res Commun 2015; 468: 79–85. 26545779 10.1016/j.bbrc.2015.10.161

[bib5] Zhan M, Zhao X, Wang H, Chen W, Xu S, Wang W et al. miR-145 sensitizes gallbladder cancer to cisplatin by regulating multidrug resistance associated protein 1. Tumour Biol 2016; 37: 10553–10562. 26852750 10.1007/s13277-016-4957-6

[bib6] Li XX, Dong Y, Wang W, Wang HL, Chen YY, Shi GY et al. Emodin as an effective agent in targeting cancer stem-like side population cells of gallbladder carcinoma. Stem Cells Dev 2013; 22: 554–566. 22974371 10.1089/scd.2011.0709 PMC3564469

[bib7] He L, Hannon GJ. MicroRNAs: small RNAs with a big role in gene regulation. Nat Rev Genet 2004; 5: 522–531. 15211354 10.1038/nrg1379

[bib8] Xuan Y, Yang H, Zhao L, Lau WB, Lau B, Ren N et al. MicroRNAs in colorectal cancer: small molecules with big functions. Cancer Lett 2015; 360: 89–105. 25524553 10.1016/j.canlet.2014.11.051

[bib9] Chan B, Manley J, Lee J, Singh SR. The emerging roles of microRNAs in cancer metabolism. Cancer Lett 2015; 356(2 Pt A): 301–308. 25451319 10.1016/j.canlet.2014.10.011

[bib10] Wu Q, Yang Z, Nie Y, Shi Y, Fan D. Multi-drug resistance in cancer chemotherapeutics: mechanisms and lab approaches. Cancer Lett 2014; 347: 159–166. 24657660 10.1016/j.canlet.2014.03.013

[bib11] Ma MZ, Chu BF, Zhang Y, Weng MZ, Qin YY, Gong W et al. Long non-coding RNA CCAT1 promotes gallbladder cancer development via negative modulation of miRNA-218-5p. Cell Death Dis 2015; 6: e1583. 25569100 10.1038/cddis.2014.541 PMC4669740

[bib12] Martinez I, Gardiner AS, Board KF, Monzon FA, Edwards RP, Khan SA. Human papillomavirus type 16 reduces the expression of microRNA-218 in cervical carcinoma cells. Oncogene 2008; 27: 2575–2582. 17998940 10.1038/sj.onc.1210919 PMC2447163

[bib13] Zhang X, Shi H, Tang H, Fang Z, Wang J, Cui S. miR-218 inhibits the invasion and migration of colon cancer cells by targeting the PI3K/Akt/mTOR signaling pathway. Int J Mol Med 2015; 35: 1301–1308. 25760926 10.3892/ijmm.2015.2126

[bib14] Nishikawa R, Goto Y, Sakamoto S, Chiyomaru T, Enokida H, Kojima S et al. Tumor-suppressive microRNA-218 inhibits cancer cell migration and invasion via targeting of LASP1 in prostate cancer. Cancer Sci 2014; 105: 802–811. 24815849 10.1111/cas.12441 PMC4317931

[bib15] Gottesman MM, Fojo T, Bates SE. Multidrug resistance in cancer: role of ATP-dependent transporters. Nat Rev Cancer 2002; 2: 48–58. 11902585 10.1038/nrc706

[bib16] Lau E, Sedy J, Sander C, Shaw MA, Feng Y, Scortegagna M et al. Transcriptional repression of IFNβ1 by ATF2 confers melanoma resistance to therapy. Oncogene 2015; 34: 5739–5748. 25728676 10.1038/onc.2015.22 PMC4558399

[bib17] Huang B, Fu SJ, Fan WZ, Wang ZH, Chen ZB, Guo SJ et al. PKCepsilon inhibits isolation and stemness of side population cells via the suppression of ABCB1 transporter and PI3K/Akt, MAPK/ERK signaling in renal cell carcinoma cell line 769 P. Cancer Lett 2016; 376: 148–154. 27037060 10.1016/j.canlet.2016.03.041

[bib18] Griffiths-Jones S, Saini HK, van Dongen S, Enright AJ. miRBase: tools for microRNA genomics. Nucleic Acids Res 2008; 36: D154–D158. 17991681 10.1093/nar/gkm952 PMC2238936

[bib19] Krech T, Scheuerer E, Geffers R, Kreipe H, Lehmann U, Christgen M. ABCB1/MDR1 contributes to the anticancer drug-resistant phenotype of IPH-926 human lobular breast cancer cells. Cancer Lett 2012; 315: 153–160. 22118813 10.1016/j.canlet.2011.09.038

[bib20] Lee SW, Lee YL, Lee YJ, Park SY, Kim IS, Choi TH et al. Enhanced antitumor effects by combination gene therapy using *MDR1* gene shRNA and HSV1-tk in a xenograft mouse model. Cancer Lett 2010; 291: 83–89. 19896764 10.1016/j.canlet.2009.10.002

[bib21] Chiu KL, Kuo TT, Kuok QY, Lin YS, Hua CH, Lin CY et al. ADAM9 enhances CDCP1 protein expression by suppressing miR-218 for lung tumor metastasis. Sci Rep 2015; 5: 16426. 26553452 10.1038/srep16426 PMC4639752

[bib22] Liu Y, Cai Q, Bao PP, Su Y, Cai H, Wu J et al. Tumor tissue microRNA expression in association with triple-negative breast cancer outcomes. Breast Cancer Res Treat 2015; 152: 183–191. 26062749 10.1007/s10549-015-3460-x PMC4484742

[bib23] Mathew LK, Skuli N, Mucaj V, Lee SS, Zinn PO, Sathyan P et al. miR-218 opposes a critical RTK-HIF pathway in mesenchymal glioblastoma. Proc Natl Acad Sci USA 2014; 111: 291–296. 24368849 10.1073/pnas.1314341111 PMC3890843

[bib24] He X, Xiao X, Dong L, Wan N, Zhou Z, Deng H et al. MiR-218 regulates cisplatin chemosensitivity in breast cancer by targeting BRCA1. Tumour Biol 2015; 36: 2065–2075. 25394901 10.1007/s13277-014-2814-z

[bib25] Wu DW, Cheng YW, Wang J, Chen CY, Lee H. Paxillin predicts survival and relapse in non-small cell lung cancer by microRNA-218 targeting. Cancer Res 2010; 70: 10392–10401. 21159652 10.1158/0008-5472.CAN-10-2341

[bib26] Jiang Z, Song Q, Zeng R, Li J, Li J, Lin X et al. MicroRNA-218 inhibits EMT, migration and invasion by targeting SFMBT1 and DCUN1D1 in cervical cancer. Oncotarget 2016; 7: 45622–45636. 27285984 10.18632/oncotarget.9850 PMC5216747

[bib27] Chen Z, Shi T, Zhang L, Zhu P, Deng M, Huang C et al. Mammalian drug efflux transporters of the ATP binding cassette (ABC) family in multidrug resistance: a review of the past decade. Cancer Lett 2016; 370: 153–164. 26499806 10.1016/j.canlet.2015.10.010

[bib28] Livak KJ, Schmittgen TD. Analysis of relative gene expression data using real-time quantitative PCR and the 2(-Delta Delta C(T)) Method. Methods 2001; 25: 402–408. 11846609 10.1006/meth.2001.1262

[bib29] Agarwal V, Bell GW, Nam JW, Bartel DP. Predicting effective microRNA target sites in mammalian mRNAs. Elife 2015; 4: e05005. 10.7554/eLife.05005 PMC4532895 26267216

[bib30] Miranda KC, Huynh T, Tay Y, Ang YS, Tam WL, Thomson AM et al. A pattern-based method for the identification of microRNA binding sites and their corresponding heteroduplexes. Cell 2006; 126: 1203–1217. 16990141 10.1016/j.cell.2006.07.031

[bib31] Kertesz M, Iovino N, Unnerstall U, Gaul U, Segal E. The role of site accessibility in microRNA target recognition. Nat Genet 2007; 39: 1278–1284. 17893677 10.1038/ng2135

[bib32] Betel D, Koppal A, Agius P, Sander C, Leslie C. Comprehensive modeling of microRNA targets predicts functional non-conserved and non-canonical sites. Genome Biol 2010; 11: R90. 20799968 10.1186/gb-2010-11-8-r90 PMC2945792

[bib33] Tomayko MM, Reynolds CP. Determination of subcutaneous tumor size in athymic (nude) mice. Cancer Chemother Pharmacol 1989; 24: 148–154. 2544306 10.1007/BF00300234

