# Supplementary material for: miR-218-5p restores sensitivity to gemcitabine through PRKCE/MDR1 axis in gallbladder cancer
Source: Cell Death Dis. 2017 May 11;8(5):e2770–. doi: 10.1038/cddis.2017.178 (PMC5520703; doi:10.1038/cddis.2017.178)
Supplement: Supplementary Information [file cddis2017178x1.doc]

**Supplement materials**

**The scoring system for immunohistochemistry and in situ hybridization (ISH)**

The scoring system was a semi-quantitative method that is based upon the staining intensity (I) and the proportion of tumor cells stained quantity (q) to obtain a final score (Q) defined as the product of I × q. The scoring system for I was: 0 = negative, 1 = low, 2 = moderate, 3 = intense immunostaining; for q was: 0 = negative, 1 = 1 - 9% positive, 2 = 10 - 39% positive, 3 = 40 - 69% positive, 4 = 70 - 100% positive cells. Scoring was performed by two independent pathologists.


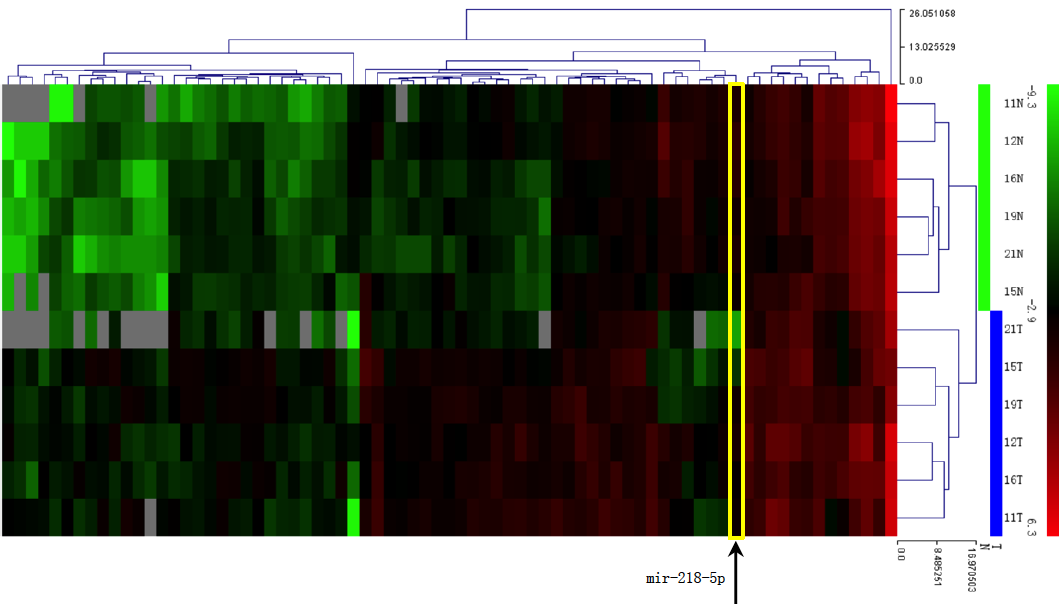


**Figure 1** Heat map displaying miRNAs with 2-fold or more differential expression between GBC and CNG tissues.


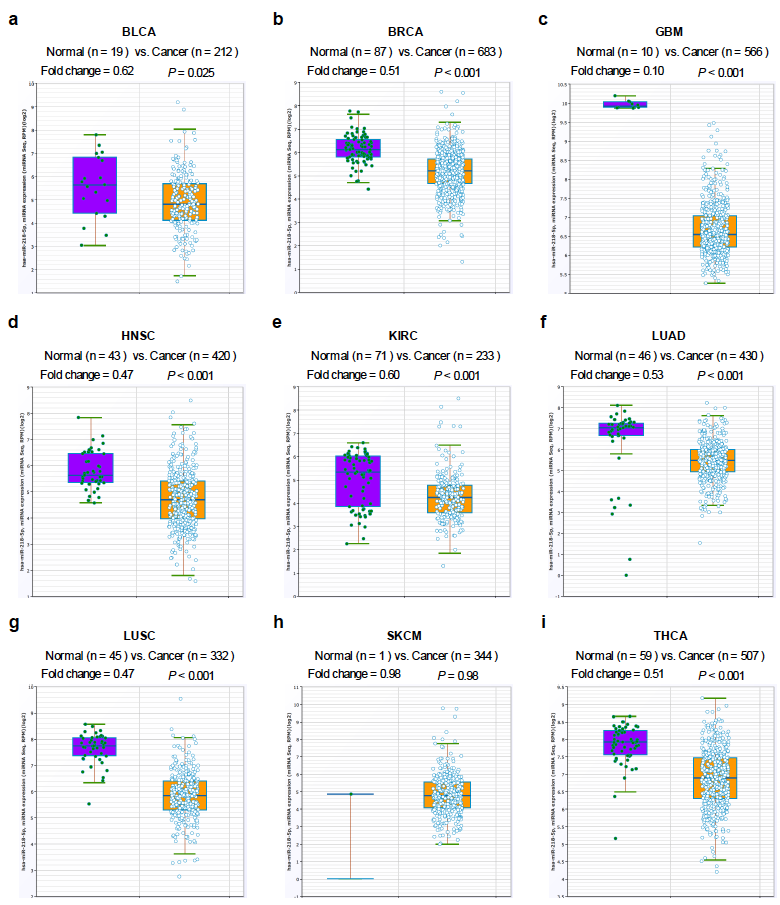


**Figure 2** miR-218-5p expression in nine kinds of cancer from starBase v2.0 Pan-cancer Project database. Urothelial bladder cancer, BLCA; Breast cancer, BRCA; Glioblastoma multiforme, GBM; Head and neck squamous cell carcinoma, HNSC; Clear cell kidney carcinoma, KIRC; Lung adenocarcinoma, LUAD; Lung squamous cell carcinoma, LUSC; Cutaneous melanoma, SKCM; Papillary thyroid carcinoma, THCA.

**
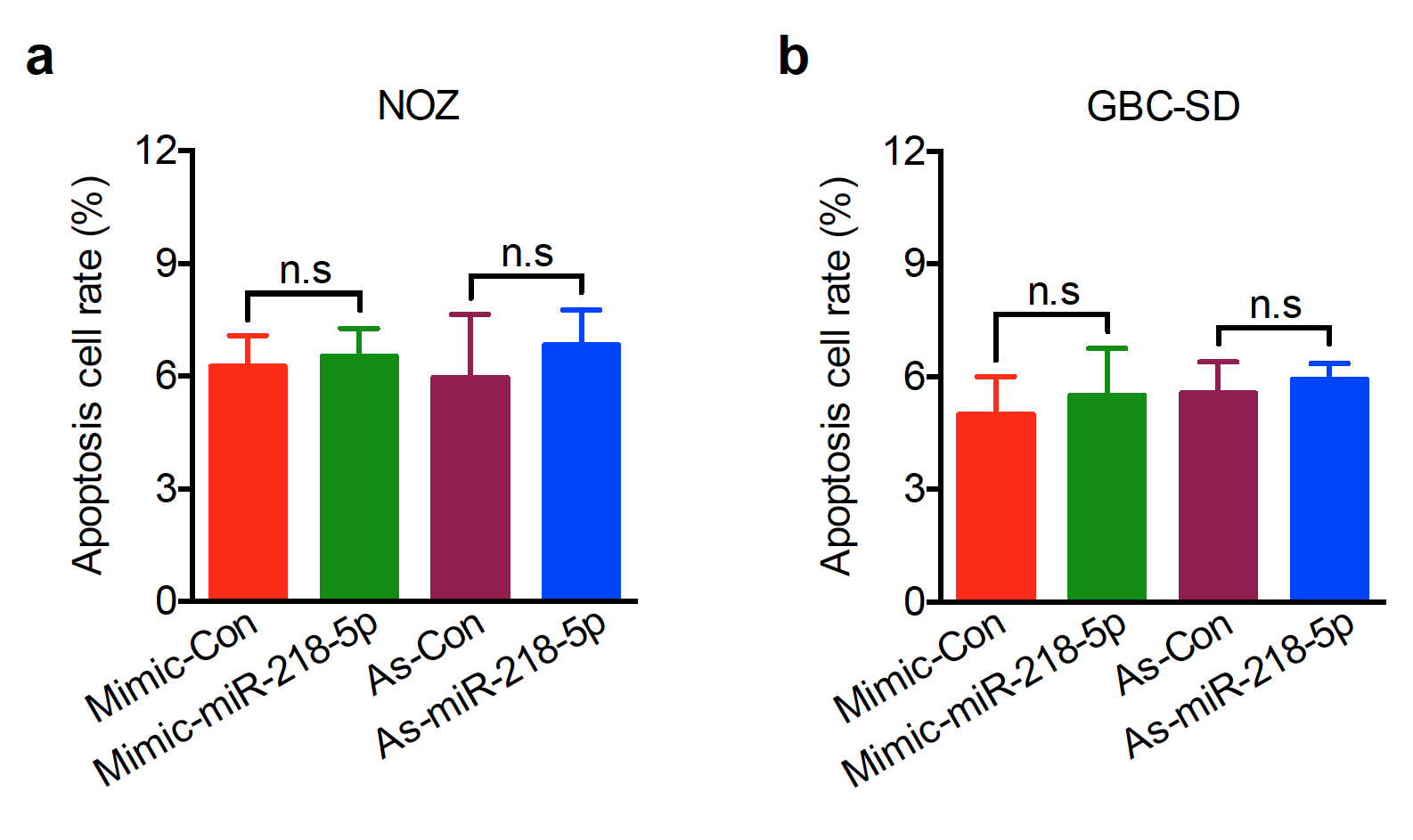
**

**Figure 3** The effect of miR-218-5p on GBC cells without gemcitabine treatment. **(a-b)** Flow cytometric analysis of Annexin V/PI staining used to quantify apoptosis rate of GBC cells transfected with miR-218-5p mimic or antagomir. n.s, no significant.

**Table 1. Significant change of microRNA expression in microarray data**

| microRNA ID | Fold change | *P*  *value* | microRNA ID | Fold change | *P*  *value* | microRNA ID | Fold change | *P*  *value* |
| --- | --- | --- | --- | --- | --- | --- | --- | --- |
| Down-regulation in GBC | | | | | | | | |
| miR-125b-5p | 0.26 | 0.008 | miR-376a-3p | 0.29 | 0.019 | miR-143-5p | 0.30 | 0.022 |
| miR-4462 | 0.31 | 0.049 | miR-136-3p | 0.32 | 0.039 | miR-100-5p | 0.35 | 0.004 |
| miR-136-5p | 0.37 | 0.027 | **miR-218-5p** | 0.41 | 0.012 | miR-376c-3p | 0.42 | 0.025 |
| miR-4324 | 0.42 | 0.001 | let-7c-3p | 0.43 | 0.019 | miR-154-5p | 0.44 | 0.029 |
| miR-193a-5p | 0.46 | 0.001 | miR-127-3p | 0.47 | 0.002 | miR-99a-3p | 0.49 | 0.014 |
| Up-regulation in GBC | | | | | | | | |
| miR-4520b-3p | 17.66 | 0.011 | miR-4280 | 10.80 | 0.049 | miR-614 | 8.96 | 0.031 |
| miR-3193 | 8.87 | 0.027 | miR-618 | 6.55 | 0.018 | miR-450b-5p | 6.21 | 0.015 |
| miR-3145-5p | 5.85 | 0.017 | miR-4275 | 5.55 | 0.022 | miR-3174 | 5.43 | 0.010 |
| miR-5694 | 5.40 | 0.030 | miR-431-3p | 5.33 | 0.008 | miR-183-5p | 5.00 | 0.011 |
| miR-548m | 4.55 | 0.042 | miR-3186-3p | 4.49 | 0.014 | miR-4735-5p | 4.30 | 0.012 |
| miR-3615 | 4.26 | 0.031 | miR-20b-5p | 3.90 | 0.024 | miR-671-3p | 3.90 | 0.027 |
| miR-5196-3p | 3.37 | 0.030 | miR-550a-3p | 3.35 | 0.010 | miR-29b-1-5p | 3.34 | 0.007 |
| miR-659-5p | 3.23 | 0.035 | miR-933 | 3.02 | 0.035 | miR-3064-3p | 2.98 | 0.005 |
| miR-10a-3p | 2.91 | 0.014 | miR-642b-3p | 2.83 | 0.049 | miR-3664-3p | 2.74 | 0.039 |
| miR-3152-5p | 2.73 | 0.033 | miR-15a-3p | 2.72 | 0.024 | miR-4738-3p | 2.61 | 0.002 |
| miR-450a-2-3p | 2.59 | 0.037 | miR-34c-5p | 2.53 | 0.035 | miR-450a-5p | 2.51 | 0.040 |
| miR-2681-3p | 2.47 | 0.011 | miR-345-5p | 2.43 | 0.015 | miR-4448 | 2.38 | 0.009 |
| miR-4490 | 2.34 | 0.019 | miR-3618 | 2.27 | 0.033 | miR-4758-3p | 2.26 | 0.045 |
| miR-589-5p | 2.24 | 0.033 | miR-3622a-3p | 2.23 | 0.009 | miR-4650-3p | 2.22 | 0.035 |
| miR-3649 | 2.19 | 0.035 | miR-647 | 2.18 | 0.012 | miR-548as-3p | 2.14 | 0.040 |
| miR-130b-5p | 2.14 | 0.033 | miR-3622b-3p | 2.13 | 0.019 | miR-1261 | 2.11 | 9×10^-4^ |
| let-7a-2-3p | 2.07 | 0.013 | miR-4503 | 2.05 | 0.018 | miRPlus-B1114 | 2.05 | 0.020 |
| miR-4632-3p | 2.04 | 0.040 | miR-4694-3p | 2.03 | 0.013 | miR-3907 | 2.01 | 0.018 |

**Table 2. Association of miR-218-5p** expression with the clinic characteristics of GBC

| Characteristics | No. of patients (n=82) | miR-218-5p  Low expression (n=42) | miR-218-5p  High expression (n=40) | χ^2^ | *P value* |
| --- | --- | --- | --- | --- | --- |
| Gender |  |  |  |  |  |
| Female, n (%) | 55(67.1) | 30(71.4) | 25(62.5) | 0.740 | 0.390 |
| Male, n (%) | 27(32.9) | 12(28.6) | 15(37.5) |
| Age, y |  |  |  |  |  |
| ≥ 60, n (%) | 64(78.0) | 32(76.2) | 32(80.0) | 0.174 | 0.677 |
| < 60, n (%) | 18(22.0) | 10(23.8) | 8(20.0) |
| Gallstone status |  |  |  |  |  |
| Yes, n (%) | 67(81.7) | 36(85.7) | 31(77.5) | 0.925 | 0.336 |
| No, n (%) | 15(18.3) | 6(14.3) | 9(22.5) |
| CA19-9, U/ml |  |  |  |  |  |
| ≥ 37, n (%) | 49(59.8) | 26(61.9) | 23(57.5) | 0.165 | 0.684 |
| < 37, n (%) | 33(40.2) | 16(38.1) | 17(42.5) |
| Tumor size, cm |  |  |  |  |  |
| ≥ 5, n (%) | 53(64.6) | 30(71.4) | 23(57.5) | 1.739 | 0.187 |
| < 5, n (%) | 29(35.4) | 12(28.6) | 17(42.5) |
| TNM stage |  |  |  |  |  |
| Ⅰ,Ⅱ | 31(37.8) | 15(35.7) | 16(40.0) | 0.160 | 0.689 |
| Ⅲ,Ⅳ | 51(62.2) | 27(64.3) | 24(60.0) |

**Table 3. Primers of Q-PCR**

| Genes (*Homo sapiens*) | Primers | Sequences |
| --- | --- | --- |
|  | Anchor RT primer | CGACTCGATCCAGTCTCAGGGTCCGAGGTATTCGATCGAGTCGCACTTTTTTTTTTTTV |
| miR-218-5p | Forward | 5'-AACACGAACTAGATTGGTACA-3' |
|  | Reverse | 5'-AGTCTCAGGGTCCGAGGTATTC-3' |
| *U6* | Forward | 5'-CTCGCTTCGGCAGCACA-3' |
|  | Reverse | 5'-AACGCTTCACGAATTTGCGT-3' |
| *MDR1* | Forward | 5'-CTTCCAAAATTTCACGTCTTGGT-3' |
|  | Reverse | 5'-ACTGACAGTTGGTTTCTTTTCCT-3’ |
| *MRP1* | Forward | 5'-CCGCTCTGGGACTGGAATG-3' |
|  | Reverse | 5'-ATGTAGCCTCGGTCATGTCG-3' |
| *BCRP* | Forward | 5'-AGGTCTGGATAAAGTGGCAGAC-3' |
|  | Reverse | 5'-AGCCAGTTGTAGGCTCATCC-3' |
| *PRKCE* | Forward | 5'-CGCCACTTCGAGGACTGGAT-3' |
|  | Reverse | 5'-GATGGACCCTGCGCCTGA-3' |
| *SFMBT1* | Forward | 5'-GCCTGCCTATATGCTGTGGA-3' |
|  | Reverse | 5'-CCATTTTGCAAACGTGTGTCC-3’ |
| *GAPDH* | Forward | 5'-GAAGGTGAAGGTCGGAGTC-3' |
|  | Reverse | 5'-GAAGATGGTGATGGGATTTC-3' |
